# Thinking While Walking: Experienced High-Heel Walkers Flexibly Adjust Their Gait

**DOI:** 10.3389/fpsyg.2013.00316

**Published:** 2013-06-03

**Authors:** Sabine Schaefer, Ulman Lindenberger

**Affiliations:** Center for Lifespan Psychology, Max Planck Institute for Human Development, Berlin, Germany

**Keywords:** dual-task, expertise, cognition, motor skills, gait

## Abstract

Theories of motor-skill acquisition postulate that attentional demands of motor execution decrease with practice. Hence, motor experts should experience less attentional resource conflict when performing a motor task in their domain of expertise concurrently with a demanding cognitive task. We assessed cognitive and motor performance in high-heel experts and novices who were performing a working memory task while walking in gym shoes or high heels on a treadmill. Surprisingly, neither group showed lower working memory performance when walking than when sitting, irrespective of shoe type. However, high-heel experts adapted walking regularity more flexibly to shoe type and cognitive load than novices, by reducing the variability of time spent in the single-support phase of the gait cycle in high heels when cognitively challenged. We conclude that high-heel expertise is associated with more flexible adjustments of movement patterns. Future research should investigate whether a more demanding walking task (e.g., wearing high heels on uneven surfaces and during gait perturbations) results in expertise-related differences in the simultaneous execution of a cognitive task.

## Introduction

Many women all over the world wear high heels, but some might recall that their first attempts to wear those shoes have been rather challenging. According to theories of motor-skill learning (e.g., Fitts and Posner, 1967), the initial stages of learning a new skill require a lot of attention. This investment of attentional resources is even more pronounced when the motor task involves some threat to balance, especially in older and balance-impaired participants (Li et al., 2001; see Woollacott and Shumway-Cook, 2002, for a review) or children (Schaefer et al., 2008). With increasing levels of practice and expertise, however, the amount of attention needed for motor-skill execution diminishes. Well-practiced motor skills should therefore lead to less interference with an attention-demanding cognitive task than motor skills that are new and not well-practiced. In a study by Beilock et al. (2002b), experienced golf players performed the secondary cognitive task more successfully than novices while putting. When disrupting the mechanisms of skill execution by using a “funny putter,” the putting accuracy of experienced golfers was reduced, and the performance of the concurrent cognitive task also suffered. Beilock and colleagues conclude that expertise leads to proceduralized control that does not require constant attention. Vuillerme and Nougier (2004) report similar findings in gymnasts.

Once skills are automatized through extensive practice, however, focusing one’s attention on skill execution can even lead to performance decrements. Beilock et al. (2002a) report that experienced golf or soccer players showed a higher putting accuracy or dribbling performance when concurrently performing a cognitive task, as compared to a condition in which they focused their attention exclusively on the step-by-step execution of the motor task. Similar findings have been obtained in adult age-comparative settings involving the motor tasks of balancing (Huxhold et al., 2006) and walking on a treadmill (Lövdén et al., 2008) in combination with cognitive tasks of different difficulty levels.

The current study aimed at investigating a dual-task situation with high-heeled gait as the motor task. Wearing high heels has been shown to change various gait and posture characteristics by, for example, increasing trunk and knee flexion angles and by leading to more asynchronous joint actions of the lower extremities (Ebbeling et al., 1994; Lee et al., 2001). Wearing high heels does involve a threat to balance, since it increases the likelihood of loosing one’s balance when slipping (Manning and Jones, 1995; Blachette et al., 2011), and the resulting falls can lead to severe injuries (Foster et al., 2012). In a study by Opila-Correia (1990), experience in wearing high heels increased the extent of knee flexion during the stance phase of high-heeled gait. In contrast, Ebbeling et al. (1994) did not observe any reliable differences in the gait characteristics of experienced and inexperienced wearers. In any case, wearing high heels is assumed to require some attention, at least in inexperienced wearers. Therefore, in a demanding dual-task situation, we expected experienced wearers of high heels to keep up their cognitive performance more efficiently than inexperienced persons when walking in high heels. Concerning walking characteristics, we analyzed the variability of different temporal-spatial gait parameters (velocity, stride time, stride length, cadence, and time spent in the single-support phase of the gait cycle). Dual-task situations have often been shown to result in increased walking variability, which in turn leads to an increased fall risk, especially in older adults (Callisaya et al., 2009; Montero-Odasso et al., 2012; Yogev-Seligmann et al., 2013). Younger adults, however, can reduce their gait variability under cognitive load (Lövdén et al., 2008). Whether individuals can stabilize their gait under cognitive load might depend on the available resources, such that being young or dealing with a well-trained task results in a more flexible adaptation of walking patterns to task demands. We predicted gait to become less variable under demanding and potentially threatening conditions, specifically when walking in high heels with a cognitive load, and especially in the experts. Differences between experienced and inexperienced high-heel wearers are expected to emerge when a secondary cognitive task is introduced because increases in task difficulty have been shown to lead to performance decrements of previously automatized motor tasks (e.g., Huxhold et al., 2006). We expected that high-heel experts would react to these challenges more flexibly than novices.

To test theses predictions, we recruited middle-aged women with different amounts of experience in wearing high heels. In a within-subject design, we asked every woman to walk on a treadmill wearing high heels and gym shoes, both under single-task conditions, and while concurrently performing a demanding working memory task. For the cognitive task, the single-task conditions consisted of performing the working memory task while sitting on a chair.

## Materials and Methods

### Participants

We recruited 48 women between 40 and 50 years of age in Berlin, Germany, who either reported to wear high-heeled shoes very frequently in everyday life (“experts,” *n* = 24), or who reported hardly ever wearing them (“novices,” *n* = 24). Experts needed to be wearing high heels at least three times a week, for at least 10 consecutive years until today, for at least 4 h on each occasion. The heels of the shoes had to be at least 6 cm high. For novices, the maximum experience that was allowed was having worn high heels for less than 20 times in the last 10 years, and for less than 2 times in the last 3 months, with a maximum duration of each episode of less than 4 h. Women who reported feeling pain when wearing high heels in the past or who reported medical conditions that might render walking on a treadmill problematic (e.g., dizziness, heart attacks or strokes, joint injuries) were excluded from participation. Table 1 reports the characteristics of the two groups. Experts and novices did not differ in age, walking speeds, or cognitive covariates, indicating that there were no reasons to assume that one of the groups was likely to outperform the other in the demanding working memory task. However, participants walked faster in gym shoes than in high heels, *t* (47) = −5.79, *p* < 0.001. The number of participants who had any experience with treadmill walking was larger in the expert group than in the novices. However, treadmill experience did not influence the preferred walking speeds in high heels or gym shoes on the treadmill, nor any of the walking parameters reported in the Section “Results.” The Ethics Committee of the MPI for Human Development approved of the study. Data collection was conducted conforming to the Declaration of Helsinki. Informed consent was obtained from all subjects and is archived by the first author. The study consisted of two sessions, each lasting for about two hours. Participants received 40 Euro for their participation.

**Table 1:** **Participant Characteristics**.

	Experts	Novices
**AGE**		
*M*	44.62	45.79
*SD*	4.18	3.27
Number of participants with/without experience with treadmill walking	13/11	7/17
**WALKING SPEED GYM SHOES (km/h)**		
*M*	3.10	3.17
*SD*	0.74	0.43
**WALKING SPEED HIGH HEELS (km/h)**		
*M*	2.87	2.63
*SD*	0.68	0.41
**DIGIT SYMBOL SUBSTITUTION (NUMBER OF ITEMS)**		
*M*	53.50	56.41
*SD*	10.46	12.88
**MWT-A (NUMBER OF ITEMS)**		
*M*	31.41	32.18
*SD*	2.63	4.02

*Digit Symbol Substitution and MWT-A were used as indicators of intelligence and educational background*.

### Apparatus

Participants were walking on a motorized treadmill (walking area 157 cm × 55 cm) with a handrail, and a virtual path was projected onto a big screen in front of them. Twelve reflective markers were placed on the following positions of each leg, according to the Plug-In Gait model: directly over the posterior superior illiac spine, directly over the anterior superior illiac spine, over the lower lateral 1/3 surface of the thigh, on the lateral epicondyle of the knee, on the tibial wand (over the lower 1/3 of the shank), on the ankle (on the lateral malleolus along an imaginary line that passes through the transmalleolar axis). In addition, both high heels and gym shoes had three markers attached to each shoe: on the heel, over the fourth metatarsal joint, and over the second metatarsal head. A Vicon MX motion analysis system with 10 near infrared cameras, recording 200 frames per second (fps), tracked the position of the markers. A commercially provided software (Vicon Nexus, v.1.2) was used to compute motion data. Motion data was pre-processed by manually filling the gaps when markers had not been recognized automatically by the system. Unfortunately, there were many gaps in the recordings of the leg markers, which were too large to be filled manually. Therefore, only the shoe markers were used for motion analyses. A MATLAB script was used to calculate the variability of different spatio-temporal gait parameters, resulting in reliable measures (Cronbach’s Alpha = 0.878). While walking, participants were always secured with a safety harness and a safety cord for emergency stops of the treadmill, but neither falls nor treadmill emergency stops occurred throughout the study. Depending on the condition, participants were either wearing gym shoes or high-heeled shoes (heel height 6.1 cm, area of the heel 4 cm^2^) while walking on the treadmill. Both types of shoes were standard exemplars, bought in a local shoe shop. We provided our participants with the same type of shoe for all possible sizes.

### Experimental tasks

#### N-back

A series of 40 numbers ranging from 1 to 9 were presented via loudspeakers, with an average inter-stimulus interval (ISI) of 2000 ms. The ISIs were jittered between 1775 and 2225 ms to prevent periodic coordination of gait patterns with cognitive task performance. Participants were instructed to say “yes” whenever the currently presented digit was identical to the digit presented *n* positions earlier (e.g., for n-back3, the bold digits of the following sequence are targets: 1 7 3 8 **7** 5 2 8 3 7 **8** 6 …). During testing, difficulty levels for n-back ranged from 1-back to 4-back. Only the 3-back task is reported here because it was the only condition in which ceiling and floor effects for accuracy were absent. There were nine target digits in each trial. Within each trial, there was a 25-s time interval (spanning about 13 digits) that did not include any targets. N-back single-task assessments took place while participants were sitting on a chair. Dual-task situations consisted of performing the n-back task while walking on a treadmill, either in high heels or in gym shoes. Participants received performance feedback after each trial.

#### Walking

Prior to the experimental trials of each session, participants were asked to choose a comfortable walking speed on the treadmill, which then remained constant for the rest of the session. Table 1 presents the walking speeds chosen by expertise group and shoe type. Participants were instructed to not use the handrail.

### Procedure

The study consisted of two sessions, one in high heels and the other in gym shoes, whose order was counterbalanced across participants. For walking and n-back, single-task performance was assessed at the beginning and the end of each session with one trial each to control for practice effects. Dual-task performance was assessed with two trials in the middle of the session. Single-task walking trials lasted for 90 s, of which 30 s were used for motion analyses. In the dual-task walking trials, motion capture took place in a 25-s time interval of the digit stream without any targets, in order to reduce the influence of verbalization on walking (Dault et al., 2003). The structure of the two sessions was identical except for the shoe type. In addition, the first session included some n-back practice trials and the second session included cognitive covariates, namely the Digit Symbol Substitution test measuring cognitive speed (Wechsler, 1981) and a modified version of the MWT-A measuring knowledge of vocabulary (Lehrl et al., 1991). For the Digit Symbol Substitution test, participants are presented with a piece of paper with nine symbols corresponding to the digits from 1 to 9. For the test, there are several rows of digits in random succession with empty spaces below them. Participants are instructed to fill in as many corresponding symbols as possible in 90 s. The score is the sum of correct responses. The MWT-A presents 37 sets of 5 words each. Only one of these five is a real word, the other four are pseudo-words. Participants are asked to choose the real word. This test has no time constraint. The score is the sum of correct answers. Table 1 presents the results for each expertise group. Experts and novices did not differ concerning these measures, indicating that their intelligence and educational background were comparable.

## Results

### N-back

**Figure 1:**
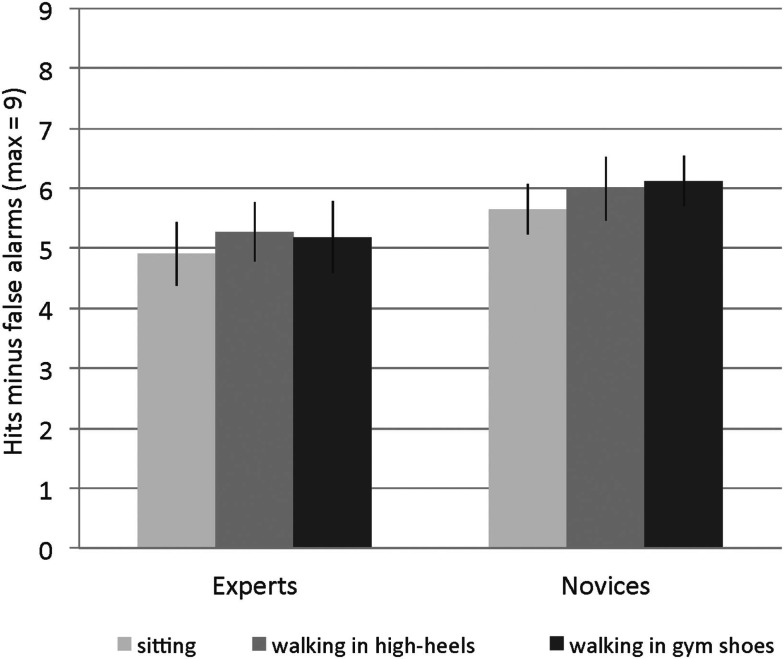
**Neither experts nor novices show performance reductions while walking in gym shoes or high heels**. Error bars = SE mean.

Figure 1 presents the results for the 3-back task. A repeated measures ANOVA with expertise group (2: experts versus novices) as between-subjects factor and single- versus dual-task performance (3: sitting, walking in high heels, walking in gym shoes) as within-subjects factor revealed no significant differences between single- and dual-task performances, *F* (2, 92) = 1.18, MSE = 1.80, *p* = 0.311, η^2^ = 0.025, and no interaction of this effect with expertise, *F* (2, 92) = 0.08, MSE = 1.80, *p* = 0.916, η^2^ = 0.002. Furthermore, expertise groups did not differ significantly in 3-back performance, *F* (1, 46) = 1.60, MSE = 14.60, *p* = 0.212, η^2^ = 0.034. Contrary to predictions, experts were not more successful than novices in keeping up their cognitive performance while walking in high heels, since both groups showed no performance reductions while walking as compared to sitting, neither in high heels nor gym shoes.

### Walking

Walking performance was assessed by calculating the variability of different spatio-temporal gait parameters using the coefficient of variation (CV). The CV is the standard deviation divided by the mean of the respective parameter [CV = (standard deviation/mean) × 100]. This measure can be interpreted as an index of gait stability, with higher variability indicating a less regular and therefore less stable and secure gait (Hausdorff, 2005). Using variability as a measure is also more appropriate than using the mean of the respective parameter, since the mean is influenced more strongly by gait velocity, which differed between gym shoes and high heels (see Table 1). Table 2 presents the CV for stride time, stride length, cadence, time spent in the single-support phase of the gait cycle (i.e., on one leg), and walking velocity, all by expertise group and shoe type. Stride length is defined as the distance covered in two steps, which is from one heel strike to the next heel strike of the same foot. Accordingly, stride time is defined as the time to perform one stride. Cadence represents the walking rate (steps per minute).

**Table 2:** **Gait parameters: coefficients of variation (%)**.

			CV velocity	CV stride time	CV stride length	CV cadence	CV single support
Experts	High heels	No load					
		*M*	2.70	3.86	3.74	4.71	3.76
		*SD*	0.77	3.73	4.58	1.94	1.97
		3-back					
		*M*	2.45	2.61	2.33	4.31	3.13
		*SD*	0.58	0.76	0.94	1.26	1.01
	Gym shoes	No load					
		*M*	2.61	2.60	2.51	4.01	3.37
		*SD*	1.04	2.04	2.38	1.73	1.54
		3-back					
		*M*	2.35	4.37	4.12	4.63	3.68
		*SD*	0.77	5.27	5.33	2.22	1.99
Novices	High heels	No load					
		*M*	2.71	2.76	2.48	4.65	3.70
		*SD*	0.61	0.64	0.82	1.10	0.79
		3-back					
		*M*	2.53	3.28	2.88	4.66	3.62
		*SD*	0.41	1.77	1.98	0.95	0.74
	Gym shoes	No load					
		*M*	2.80	2.53	2.61	3.94	3.13
		*SD*	0.81	1.55	1.92	1.08	0.85
		3-back					
		*M*	2.46	2.62	2.45	3.81	2.97
		*SD*	0.48	1.46	1.60	0.94	0.76

To assess which factors influenced gait variability, mixed-design ANOVAs with expertise group (2: experts versus novices) as between-subjects factor and shoe type (2: high heels versus gym shoes) and single- versus dual-tasking (2: walking with or without cognitive load) as within-subjects factors were conducted. The results of these analyses are summarized in Table 3.

**Table 3:** **Mixed-design ANOVA results for coefficients of variation**.

	CV velocity	CV stride time	CV stride length	CV cadence	CV single support
Shoe type	*F* (1, 46) = 0.24	*F* (1, 46) = 0.07	*F* (1, 46) = 0.03	*F* (1, 46) = 5.73	*F* (1, 46) = 2.61
	MSE = 0.34	MSE = 6.65	MSE = 7.78	MSE = 1.95	MSE = 1.31
	*p* = 0.523	*p* = 0.787	*p* = 0.867	***p* = 0.021**	*p* = 0.113
	η^2^ = 0.005	η^2^ = 0.002	η^2^ = 0.001	η^2^ = 0.111	η^2^ = 0.054
Shoe type × group	*F* (1, 46) = 0.41	*F* (1, 46) = 0.86	*F* (1, 46) = 0.28	*F* (1, 46) = 2.10	*F* (1, 46) = 4.37
	MSE = 0.34	MSE = 6.65	MSE = 7.78	MSE = 1.95	MSE = 1.31
	*p* = 0.523	*p* = 0.357	*p* = 0.598	*p* = 0.154	***p* = 0.042**
	η^2^ = 0.009	η^2^ = 0.018	η^2^ = 0.006	η^2^ = 0.044	η^2^ = 0.087
Cognitive load	*F* (1, 46) = 13.78	*F* (1, 46) = 1.15	*F* (1, 46) = 0.16	*F* (1, 46) = 0.03	*F* (1, 46) = 1.97
	MSE = 0.23	MSE = 3.27	MSE = 3.58	MSE = 1.05	MSE = 0.47
	***p* = 0.001**	*p* = 0.289	*p* = 0.693	*p* = 0.863	*p* = 0.167
	η^2^ = 0.231	η^2^ = 0.024	η^2^ = 0.003	η^2^ = 0.001	η^2^ = 0.041
Cognitive load × group	*F* (1, 46) = 0.00	*F* (1, 46) = 0.01	*F* (1, 46) = 0.00	*F* (1, 46) = 0.33	*F* (1, 46) = 0.04
	MSE = 0.23	MSE = 3.27	MSE = 3.58	MSE = 1.05	MSE = 0.47
	*p* = 0.988	*p* = 0.934	*p* = 0.976	*p* = 0.570	*p* = 0.848
	η^2^ = 0.000	η^2^ = 0.000	η^2^ = 0.000	η^2^ = 0.007	η^2^ = 0.001
Shoe type × load	*F* (1, 46) = 0.38	*F* (1, 46) = 3.74	*F* (1, 46) = 2.88	*F* (1, 46) = 3.56	*F* (1, 46) = 4.26
	MSE = 0.22	MSE = 5.44	MSE = 6.38	MSE = 0.64	MSE = 0.54
	*p* = 0.539	*p* = 0.059	*p* = 0.097	*p* = 0.065	***p* = 0.045**
	η^2^ = 0.008	η^2^ = 0.075	η^2^ = 0.059	η^2^ = 0.072	η^2^ = 0.085
Shoe type × load × group	*F* (1, 46) = 0.29	*F* (1, 46) = 6.50	*F* (1, 46) = 6.02	*F* (1, 46) = 6.36	*F* (1, 46) = 5.83
	MSE = 0.22	MSE = 5.44	MSE = 6.38	MSE = 0.64	MSE = 0.54
	*p* = 0.592	***p* = 0.014**	***p* = 0.018**	***p* = 0.016**	***p* = 0.020**
	η^2^ = 0.006	η^2^ = 0.124	η^2^ = 0.116	η^2^ = 0.120	η^2^ = 0.113
Group	*F* (1, 46) = 0.37	*F* (1, 46) = 1.27	*F* (1, 46) = 1.00	*F* (1, 46) = 0.20	*F* (1, 46) = 0.18
	MSE = 1.24	MSE = 11.93	MSE = 15.55	MSE = 5.08	MSE = 4.54
	*p* = 0.545	*p* = 0.266	*p* = 0.322	*p* = 0.654	*p* = 0.676
	η^2^ = 0.008	η^2^ = 0.027	η^2^ = 0.021	η^2^ = 0.004	η^2^ = 0.004

*All p-values smaller than p = 0.05 are printed in bold*.

**Figure 2:**
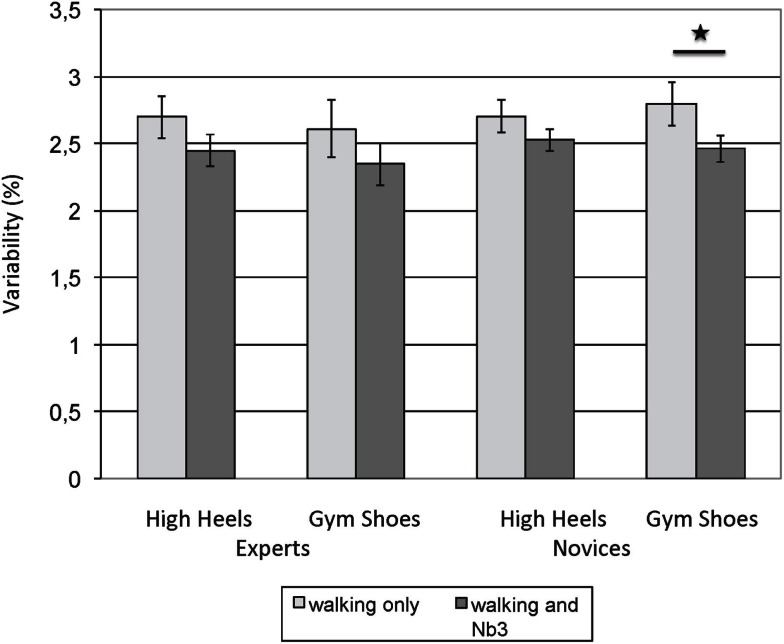
**Variability of walking velocity (coefficient of variation): experts and novices reduce their gait variability under cognitive load, independent of shoe type**. Error bars = SE mean.

The pattern of results was not uniform across the different gait parameters. For walking velocity, only the main effect of cognitive load reached significance. A follow-up *t*-test comparing the variability of velocity under single-task conditions to the variability under cognitive load (averaging across expertise group and shoe type) indicates that this main effect reflected a reduction of variability under cognitive load, *t* (47) = 3.75, *p* = 0.000. When single- and dual-task performances are contrasted within each group and for each shoe type separately, the respective *t*-test only reaches significance for novices in gym shoes. Figure 2 depicts these results graphically.

**Figure 3:**
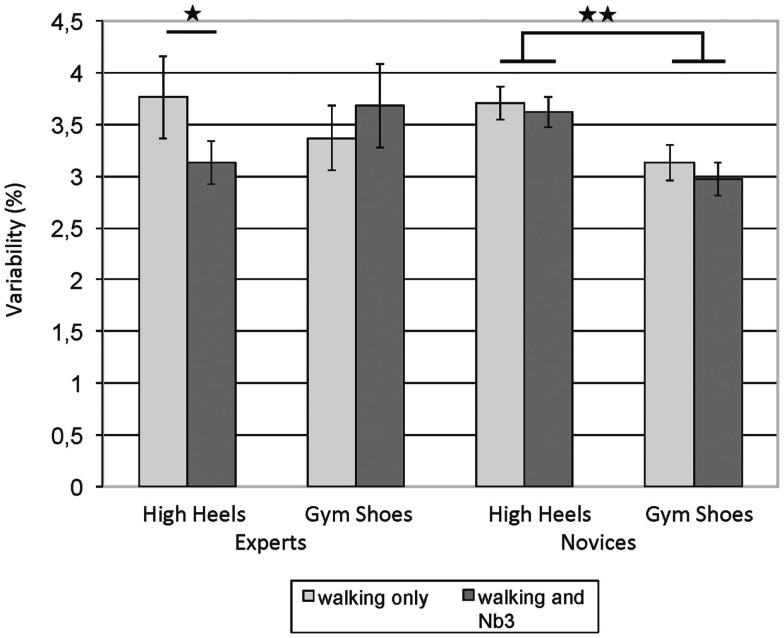
**Variability of time spent in single support (coefficient of variation): high-heel experts flexibly adjust their gait according to cognitive load when wearing high heels, novices show little changes in both shoe types when cognitively challenged**. Error bars = SE mean.

We also observed a main effect of shoe type for the variability of cadence, caused by a more variable gait in high heels as compared to gym shoes, averaging over cognitive load and expertise group [*t* (47) = 2.37, *p* = 0.022]. In the analyses for stride time, stride length, cadence, and time spent in single-support, the three-way interaction of shoe type, cognitive load and expertise group was statistically reliable, *p* < 0.05. Follow-up *t*-tests comparing single- versus dual-task performance within each expertise group and for each shoe type separately show that the interaction was primarily due to experts increasing their gait variability under cognitive load when walking in gym shoes for the parameters stride time [*t* (23) = −2.20, *p* = 0.038] and cadence [*t* (23) = −2.63, *p* = 0.015]. For time spent in single support, however, the interaction was due to experts *decreasing* their variability in high heels under cognitive load, *t* (23) = 2.22, *p* = 0.037, as shown in Figure 3. All other *t*-tests did not reach statistical significance. In addition, for time spent in single support, the interaction of shoe type and expertise group was reliable. Paired samples *t*-tests comparing the gait variability in gym shoes versus high heels, averaging over cognitive load but separately for each expertise group revealed that novices showed more variability in high heels as compared to gym shoes, *t* (23) = 3.53, *p* = 0.002, while this difference did not reach significance in the experts.

In sum, contrary to expectations, experts did not show higher levels of performance in a working memory task than novices when performing the task while wearing high heels. At the same time, experts adjusted their walking variability more flexibly to the requirements of the specific task conditions, while novices showed fewer adjustments.

## Discussion

There were no differences between high-heel experts and novices in their cognitive performances while walking. Both groups managed to show comparable scores in a demanding working memory task when sitting on a chair or when walking on a treadmill in gym shoes or high heels.

According to theories of motor learning (e.g., Fitts and Posner, 1967), the amount of attention that needs to be invested into a motor task declines with increasing practice. High-heel experts were therefore assumed to show higher cognitive performances when walking in high heels than novices. Walking in itself, however, is usually highly automatized and does not necessarily lead to performance decrements in cognition (Lövdén et al., 2008; Schaefer et al., 2010). It is possible that the predicted effects did not occur in the current study because high-heeled walking was not difficult enough. For example, the heels of the shoes were not extremely high (6.1 cm), and the area of the heel (4 cm^2^) was rather large, providing more stability to participants than a more extreme shoe version. In addition, participants were allowed to choose their preferred speed for each walking condition. Walking also did not include any perturbations, which do happen in real-life walking (e.g., when stepping over an obstacle or when dealing with different surfaces). Perhaps expertise effects would emerge under more “realistic” walking conditions. Another possibility is that people adapt very quickly to walking in high heels, such that the time needed to find their preferred speed on the treadmill already provided sufficient practice, even for novices.

Results for the walking task revealed a rather complex pattern. Although the number of participants with treadmill experience was larger in the expert group, treadmill experience did not lead to faster walking speeds in gym shoes or high heels on the treadmill, and it did not influence gait variability in any of the parameters under investigation. It is therefore unlikely that different levels of treadmill experience influenced the findings.

There were changes with expertise for single- versus dual-task walking variability, depending on the type of shoe and on the exact gait parameter under investigation. For time spent in single support, novices showed more variability in their gait in high heels than in gym shoes, indicating that their high-heeled gait was less stable and secure. For gait velocity, walking became more stable under cognitive load. For cadence, there was higher variability in high-heeled gait as compared to gym shoes, irrespective of cognitive load and expertise. The most consistent pattern, which was found in four out of five analyses, was the three-way interaction of shoe type, cognitive load, and expertise group. It was caused by experts showing more change in their gait variability when cognitively challenged, either by reducing their variability in high heels (time spent in single support) or by increasing their variability in gym shoes (stride time and cadence). Reductions of gait variability are usually assumed to represent a more stable and more secure gait (Hausdorff, 2005). The experts’ behavior in high heels for the variability of time spent in single support can therefore be considered adaptive, since high-heeled gait might increase the likelihood of falling and getting injured (e.g., by twisting one’s ankle; Foster et al., 2012). In gym shoes, there is a smaller likelihood of loosing balance and of hurting oneself compared to high heels, such that the observed increase in walking variability for the experts might not be as problematic. It also indicates that wearing gym shoes is a less well-practiced task for women who spent a lot of time wearing high heels (Cronin et al., 2012).

Taken together, the current study did not find the predicted pattern of dual-task related cognitive benefits for a group of experts as compared to novices walking in high heels while performing a cognitive task. Neither group showed lower working memory performance when walking than when sitting, irrespective of shoe type. However, high-heel experts adapted their walking more flexibly to shoe type and cognitive load than novices, by reducing the variability of time spent in the single-support phase of the gait cycle in high heels when cognitively challenged. In high-heeled gait, expertise therefore leads to more flexible adjustments of movement patterns. Parametric difficulty variations of the motor and the cognitive task are needed to gain a more complete picture of the interactions between motor expertise and cognitive performance. However, the present study indicates that wearing high heels does not necessarily disrupt your thinking, even if you have little experience with those shoes.

## Conflict of Interest Statement

The authors declare that the research was conducted in the absence of any commercial or financial relationships that could be construed as a potential conflict of interest.
